# Unilateral Asterixis in Arm and Leg Caused by Internal Capsula Stroke

**DOI:** 10.1155/2018/3946380

**Published:** 2018-01-18

**Authors:** Katharina Feil, Marion Huber, Nicolina Goldschagg, Lars Kellert

**Affiliations:** ^1^Department of Neurology, Ludwig Maximilians University, Munich, Germany; ^2^German Center for Vertigo and Balance Disorders, Ludwig Maximilians University, Munich, Germany; ^3^Department of Neurology, University Hospital Heidelberg, Heidelberg, Germany

## Abstract

We report an unusual clinical manifestation of ischemic stroke with acute right-sided asterixis affecting the arm as well as the leg due to a lesion in the left posterior limb of the internal capsula. After treatment with intravenous thrombolysis the patient made a good recovery. Notably, in this case unilateral asterixis affected the arm as well as the leg, resulting in postural and gait instability. In addition, damage in the basal ganglia-thalamo-cortical network, as in our patient, has to be distinguished from other supratentorial causes of acute asterixis like thalamic or frontal lobe lesions linked to the cerebello-brainstem-thalamo-frontal lobe circuits.

## 1. Introduction

Movement disorders due to cerebrovascular diseases are diverse and can differ in clinical presentation as well as pathophysiology, prognosis, and treatment [1]. Limb-shaking has been known to be a symptom of TIA, closely associated with hemodynamic impairment in the anterior circulation [2]. However, electrophysiological limb-shaking can prove to be asterixis by using surface EMG [3]. Furthermore, cases of stroke-associated asterixis in the arms as well as isolated in the leg have been reported [4, 5]. Here, we report a case of transient asterixis, documented by video, affecting both the arm and leg in a case of stroke in the posterior limb of the internal capsula.

## 2. Case Presentation

**Figure 1:**
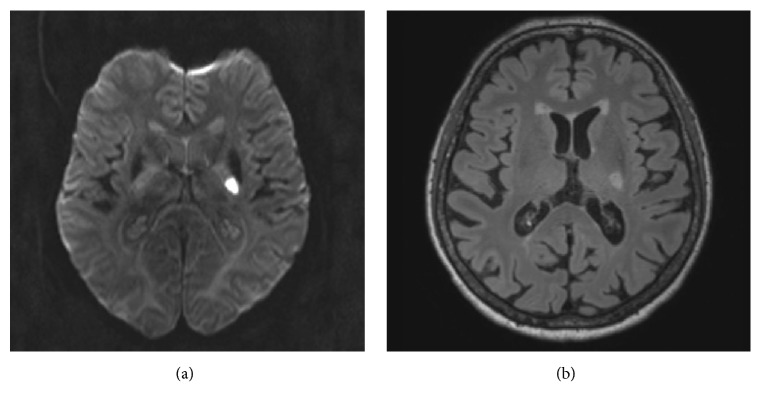
(a) Axial imaging showing diffusion restriction with a corresponding reduction in signal ADC map (not shown in figure). (b) Axial flair imaging with hyperintense infarction areal in the capsule leg on the left posterior internal capsula.

A 74-year-old right-handed woman, without any relevant medical history or any cardiovascular risk factors, was admitted to our emergency room. Clinical examination revealed no paresis (5/5 on the MRC scale), but unilateral severe asterixis of the right side and fine motor skill disturbances of the right hand were found, as well as reduced muscle tone on the right side and a positive Babinski sign (see Video 1). The cranial nerve examination, sensory examination, and deep tendon reflexes were normal and symmetrical (overall 2 points on the NIHSS). Laboratory data (including liver and renal function and ammonia) were also completely unremarkable. Cranial CT imaging at admission (55 minutes after symptom onset) including CT-perfusion showed normal imaging without artery occlusion or perfusion mismatch. The patient received intravenous thrombolysis due to suspected ischemic stroke. Within one day unilateral asterixis disappeared and only fine motor disturbances of the right hand remained at discharge. In consequence, since the clinical feature disappeared, a surface EMG examination could no longer be performed. An MRI scan showed signal change consistent with acute infarction involving the posterior limb of the left internal capsula (see Figure 1). There were no abnormal lesions in the thalamus bilaterally, the basal ganglia, or the cerebellum. The diagnostic workup of the patient's stroke included all relevant examinations and led to a diagnosis of large-artery atherosclerosis stroke (TOAST subtype 1) [6] due to hypercholesterinemia and macroangiopathy.

## 3. Discussion

Asterixis is an arrhythmic, abrupt negative myoclonus due to irregular intermittent interruption of tonic muscle contractions evoked usually in the upper limbs when the arms are outstretched [7]. It is predominantly associated with metabolic diseases and was first described in the context of hepatic encephalopathy. Hyperkinetic movement disorders, such as tremor, chorea, dystonia, and asterixis, may occur in 1–4% of ischemic stroke cases [1, 8]. Poststroke asterixis is usually unilateral and rarely bilateral and predominantly affects the upper limb and develops during the acute phase, as in our case [1]. Asterixis has been reported mainly after ischemic lesions of the basal ganglia, thalamus, frontoparietal cortex, cerebellum, and brainstem, which usually cause unilateral asterixis in the arms [1, 9]. To date there is only one case in the literature with postural and gait instability due to asterixis exclusively in the legs after ACA territory infarction [5]. In contrast to previous cases [4, 9], we report a patient with unilateral asterixis affecting both the upper and lower limb, which is very rare. Although an EMG examination to prove the brief interruptions of tonic muscle activity which characterize negative myoclonus (i.e., asterixis) was not possible because the symptoms disappeared within a short period of time after thrombolysis, the clinical presentation, as documented in the video, was typical for asterixis.

Along with the case report by Matsumoto et al. [10], we can confirm that acute stroke in the posterior area of the internal capsula can cause acute and temporary unilateral asterixis without any further motor or sensory deficiencies. The pathophysiology of unilateral asterixis occurring in internal capsula stroke remains unclear. Due to the different lesions of symptomatic unilateral asterixis, one can propose that there are cortico-subcortical pathways involving the basal ganglia, motor thalamus, cerebellum, and frontal-parietal neocortex to control postural tone, assuming that this basal ganglia-thalamo-cortical circuit may be important in adjusting postural tone and movement [8]. Furthermore, unilateral asterixis might be mediated by structural lesions in this basal ganglia-thalamo-cortical network, as in our case due to a lesion in the internal capsula. This network has to be distinguished from other supratentorial causes of acute asterixis like the lateral thalamus and frontal lobe, as these localizations are connected to the cerebello-brainstem-thalamo-frontal lobe circuits [8, 9]. In conclusion, we report a novel case with internal capsula stroke, initially starting with hemiparesis and then presenting only with asterixis contralateral to the lesion side. After intravenous thrombolysis, symptoms resolved within days with only latent hemiparesis with mild fine motor skill disturbances at discharge.

## Supplementary Materials

Supplementary Materials Video 1: clinical examination of the patient in the emergency room showing hemiasterixis involving leg and arm.
